# Analysis of Heritability Across the Clinical Phenotypes of Frontotemporal Dementia and the Frequency of the C9ORF72 in a Colombian Population

**DOI:** 10.3389/fneur.2021.681595

**Published:** 2021-08-30

**Authors:** Andrea López-Cáceres, María Velasco-Rueda, Elkin Garcia-Cifuentes, Ignacio Zarante, Diana Matallana

**Affiliations:** ^1^School of Medicine, Instituto de Genética Humana, Pontificia Universidad Javeriana, Bogotá, Colombia; ^2^Fundación Santa Fé de Bogotá, Bogotá, Colombia; ^3^School of Medicine, Departamento de Neurociencias, Unidad de neurología, Pontificia Universidad Javeriana, Bogotá, Colombia; ^4^School of Medicine, Instituto de Envejecimiento, Doctorado de Neurociencias, Psychiatry and Mental Health Department, Pontificia Universidad Javeriana, Bogotá, Colombia; ^5^Centro de Memoria y Cognición Intellectus, Hospital Universitario San Ignacio, Bogotá, Colombia

**Keywords:** C9ORF72, frontotemporal dementia, Colombia, family inheritance, heritability

## Abstract

Frontotemporal dementia (FTD) is a highly heritable condition. Up to 40% of FTD is familial and an estimated 15% to 40% is due to single-gene mutations. It has been estimated that the G4C2 hexanucleotide repeat expansions in the C9ORF72 gene can explain up to 37.5% of the familial cases of FTD, especially in populations of Caucasian origin. The purpose of this paper is to evaluate hereditary risk across the clinical phenotypes of FTD and the frequency of the G4C2 expansion in a Colombian cohort diagnosed with FTD.

**Methods:** A total of 132 FTD patients were diagnosed according to established criteria in the behavioral variant FTD, logopenic variant PPA, non-fluent agrammatic PPA, and semantic variant PPA. Hereditary risk across the clinical phenotypes was established in four categories that indicate the pathogenic relationship of the mutation: high, medium, low, and apparently sporadic, based on those proposed by Wood and collaborators. All subjects were also examined for C9ORF72 hexanucleotide expansion (defined as >30 repetitions).

**Results:** There were no significant differences in the demographic characteristics of the patients between the clinical phenotypes of FTD. The higher rate phenotype was bvFTD (62.12%). In accordance with the risk classification, we found that 72 (54.4%) complied with the criteria for the sporadic cases; for the familial cases, 23 (17.4%) fulfilled the high-risk criteria, 23 (17.4%) fulfilled the low risk criteria, and 14 (10.6%) fulfilled the criteria to be classified as subject to medium risk. C9ORF72 expansion frequency was 0.76% (1/132).

**Conclusion:** The FTD heritability presented in this research was very similar to the results reported in the literature. The C9ORF72 expansion frequency was low. Colombia is a triethnic country, with a high frequency of genetic Amerindian markers; this shows consistency with the present results of a low repetition frequency. This study provides an initial report of the frequency for the hexanucleotide repeat expansions in C9ORF72 in patients with FTD in a Colombian population and paves the way for further study of the possible genetic causes of FTD in Colombia.

## Introduction

Frontotemporal dementia (FTD), a heterogeneous neurodegenerative disorder, is a highly heritable condition with reports of a positive family history in as many as 60% of cases (1, 2). In order to estimate the heritability of the family history, some criteria have been standardized—following the Goldman score and the one proposed by Wood and collaborators—according to the number of first- and second-degree relatives affected by FTD (3, 4). These efforts suggest a disease mechanism regarding the likelihood of an identifiable genetic cause and variability across clinical phenotypes (4, 5). A strong family history and higher frequency has been found in the behavioral variant of FTD (bvFTD), but less so in the semantic variant PPA (svPPA), the logopenic variant PPA (lvPPA), and the non-fluent agrammatic PPA (nfaPPA) (5–9). The heritability of FTD with motor neuron disease (FTD-MND), and atypical parkinsonian disorders are less clear, possibly due to the number of studies reported until today (5, 10). However, the G4C2 (GGGGCC) hexanucleotide repeat expansions in the C9ORF72 gene is the most common genetic cause of ALS and FTD (11, 12), and although the expansion mechanism is uncertain, it is suggested that the cause of disease in FTD includes “gain-of-toxicity” or reduction in function of the C9ORF72 protein (13).

It has been estimated that G4C2 can explain up to 37.5% of the familial cases of FTD, in particular, in populations of Caucasian origin (14). G4C2 has also been reported as a major cause of the disease in northern Europe, mainly Finland, and in North American FTD and ALS cohorts (11, 15). C9ORF72 also accounts for a significant proportion of Australian and Spanish FTD cases (16). By contrast, the C9ORF72 repeat expansion was not present or extremely rare in patients of Native American, Pacific Islander (11), Asian (17, 18), and Middle Eastern countries (19), and China (20, 21). Very few studies on the frequency of C9ORF2 have been carried out in Latin America. The first report was in an Argentinian population, where the expansion frequency in a FTD group was similar to that reported for patients in Europe and North America (14). In a Brazilian population (22, 23), the frequencies of the mutation in pure ALS and pure FTD cases were much lower than those observed in Finnish patients (11, 24), but similar to what was found for Germany (11) and Flanders-Belgium (25). There are no data as yet on the frequency and heritability of this expansion in an FTD population in Colombia (26). As such, in this study, we expect to estimate the frequency and heritability of C9ORF72 hexanucleotide repeat expansion in a group of patients with FTD diagnosis in Colombia.

## Materials and Methods

### Population

A total of 132 patients were diagnosed with FTD according to consensus criteria for bvFTD, PPA: lvPPA, nfaPPA, and svPPA (27–29), at the Memory and Aging Clinic at the Hospital Universitario San Ignacio and Pontificia Universidad Javeriana in Bogotá, Colombia. The ethnicity of our sample could not be directly verified, but all patients are Colombian, and reported to be of Hispanic origin. This study was approved by the Ethics Committee at the same institution, and written consent was obtained from all participants and their legal representatives.

### Pedigree

Family trees of the patients with FTD diagnosis were drawn up using information provided by the patients' families and caregivers. Pedigree information was obtained using the Proband application, where at least three generations of each of the subjects were described. The heritability of the disorder was classified by a geneticist with experience in the field of neurodegenerative diseases. The classification criteria were based on those proposed by Wood and collaborators. This classification method has four categories that indicate the pathogenic relationship of the mutation: high, medium, low, and apparently sporadic. These criteria are based on the number of first- and second-degree relatives affected with the spectrum of FTD disorders or other neurodegenerative diseases (4).

### Gene Sequencing and Genotyping

Genomic: All evaluated patients had a 3-cc blood sample taken in EDTA (ethylenediaminetetraacetic acid) tubes from which the genomic DNA was extracted using the Salting Out protocol. The DNA was then quantified using a NanoDrop® ND-1000 spectrophotometer. C9ORF72 hexanucleotide expansion (defined as >30 repetitions) was analyzed and tested with repeat-primed PCR and capillary electrophoresis as previously described (30). The sizes of the PCR fragments were analyzed using GeneMapper software (Applied Biosystems, Foster City, CA).

### Statistics

A frequency distribution was performed taking into account the risk classification of the pedigrees together with phenotypic (sex, age, and diagnosis) and genotypic (presence of the C9ORF72 expansion) characteristics. For the statistical analysis, absolute and relative measures were obtained for quantitative data. Central tendency and dispersion measures were evaluated for quantitative data.

## Results

Of the 132 patients, 51.52% were males and 48.48% were females. The latter presented a lower prevalence in the low-risk group than the male group. The main age of onset was of 59 years (12 IQR) (Table 1). The higher rate phenotype was bvFTD (62.12%), followed by non-specific PPA (18.18%), svPPA (15.90%), lvPPA (3.03%), and nfaPPA (0.75%). In categorizing by genetic risk based on the Wood pedigree classification, we found that 72 (54.4%) complied with the criteria for the sporadic cases; for the familial cases, 23 (17.4%) fulfilled the criteria for being high risk; 23 (17.4%) fulfilled the criteria for low risk; and 14 (10.6%) fulfilled the criteria for medium risk. Females and males were similarly distributed in three of the risk classification groups: apparent sporadic (40/32), medium risk (8/6), and high risk (12/11). The low-risk classification included more men than women (4/19).

**Table 1:** FTD spectrum disorder pedigree categorization according to sex, age of onset, phenotype, and C9orf72 genotype.

		**Apparent sporadic**	**Low**	**Medium**	**High**	**Total**
		***n***	***n***	***n***	***n***	***n***
Sex	Female	40	4	8	12	64 (48.48%)
	Male	32	19	6	11	68 (51.51%)
Age of onset	Median (IQR)	59	57	59	63.1	59
Phenotype	bvFTD	44	14	8	16	82 (62.12%)
	PPA	11	5	3	5	24 (18.18%)
	svPPA	14	3	3	1	21 (15.90%)
	lvPPA	2	0	0	2	4 (3.0%)
	nfaPPA	1	0	0	0	1 (0.76%)
C9orf72	Presence of the expansion	1	0	0	0	1 (0.76%)

*IQR, interquartile range; bvFTD, behavioral variant frontotemporal dementia; nfaPPA, non-specific PPA; PPA, primary progressive aphasia; PPA-lv, primary progressive aphasia logopenic variant; PPA-nfv, primary progressive aphasias non-fluent variant; PPA-sv, semantic variant*.

**Figure 1:**
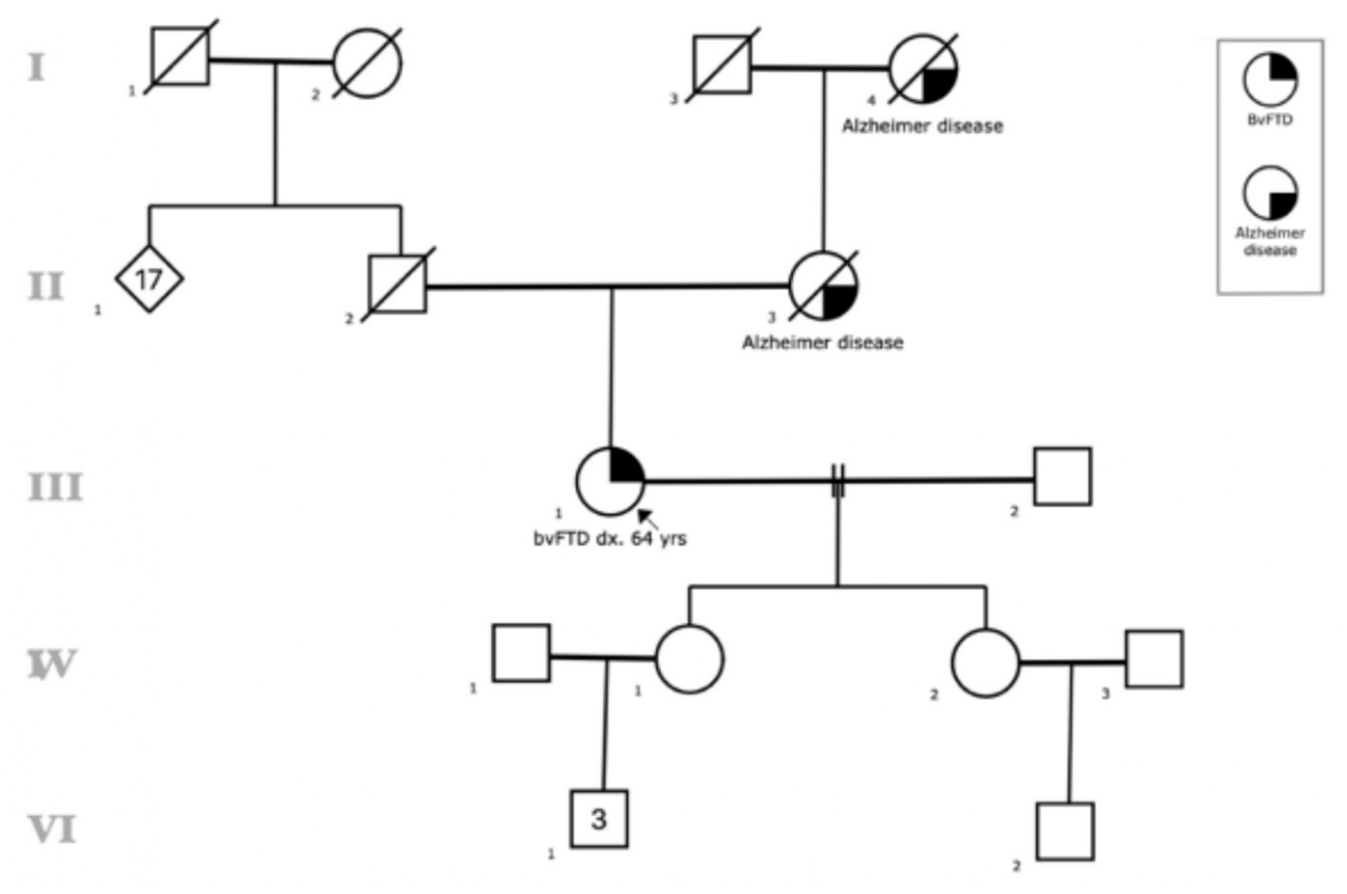
Family Pedigree of the patient with C90RF72 expansion. Circles: female, square: males. Black symbols reflect individuals affected with Alzheimer disease or bvFTD, lines represent those who are deceased. The arrow identifies the proband.

C9ORF72 expansion was observed in 0.76% (1/132) of the sample. The positive case is a female patient diagnosed with bvFTD. The family pedigree was classified as a high-risk familial case (Figure 1), and the simple brain MRI with contrast revealed moderate supratentorial cortical atrophy predominantly in frontal and temporal regions.

## Discussion

The present results show that the Colombian FTD sample data are similar to what is described in the literature regarding heritability, age of onset, and time of evolution of the disorder (31). Most of our patients exhibited the bvFTD followed by language variants (11, 32). One previous study demonstrated that bvFTD and the non-fluent/agrammatic variant of primary progressive aphasia (nfv-PPA) appeared to be more heritable than the semantic variant of primary progressive aphasia (sv-PPA) (33).

We observed no differences in the overall percentage of men and women in the study population, as has been reported in studies of populations in Argentina, southern Italy, and Brazil where the percentage of female patients has been higher (14, 23, 34). However, we note that our only case with the G4C2 expansion was presented by a woman and that our percentage of women classified as being of low heritable risk was much lower than that presented in other risk groups, which could support the hypothesis that female G4C2 repeat mutation carriers are more likely to develop cognitive or behavioral impairment (35). Given previous reports where C9ORF72 expansions have been found in non-familial cases (11), we found only one patient with the bvFTD that presented the C9ORF72 expansion from the high-risk cases, with a total frequency of 0.76% (1/132). The repeat expansions in the C9ORF72 gene is responsible for one of the FTD cases but not all FTD diagnoses in a Colombian cohort, revealing that there may be causes other than C9ORF72 to account for FTD cases in Colombia.

Wood and collaborators found C9ORF72 expansion in 25/306 (8.2%) of FTD patients, with the mutation-detection rate being highest in the low category and apparent sporadic cases (12, 24). This finding is consistent with prior reports of C9ORF72 expansion in sporadic families, and it coincides with findings from other studies (11, 36). Although we found C9ORF72 expansion in the high-risk group, we found no other patients that fulfilled the high-risk criteria and presented the expansion, supporting the importance of performing molecular analysis of this expansion in the idiopathic forms (11, 37–39).

The low frequency of the G4C2 expansion in the patient group with FTD 0.76% (1/132) is similar to what has been reported for Asian and Amerindian populations (17–21). There are even studies where no cases with this expansion 0/52 were identified (40). In Europe and North America, much higher frequencies have been established for the G4C2 expansion, with Finland and Sweden with overall frequencies of 29.33 and 20.73%, respectively, and Spain with 25.49%. Lower frequencies have been observed in Germany with 4.82% (41). In North America, C9ORF72 expansion accounted for almost 25% of familial FTD cases and 6% of sporadic cases (11). So far, only two studies have been conducted for the Latin American population, one in Argentina (14) where a frequency of expansion of 18.2% (6/33 cases) of patients with FTD was observed (14), and the other in Brazil, where a frequency of 7.1% (*n* = 67) for patients with pure familial FTD was found (23).

As it was shown before, the high frequency of the C9ORF72 expansion is associated with populations of European origin (11, 14). According to the human settlement hypothesis, Asian populations arriving through the Bering strait settled in North and South America, making the Amerindian populations very similar to the original ones and homogeneous with each other. This would support the absence of the C9ORF72 repetition in populations of Amerindian origin and this coincides with the results found for Amerindian groups in North America (11).

The populations of European ancestry with high frequencies present similar frequencies. An example of this is the Argentine population among which frequencies similar to those of European countries have been found, corroborating the Caucasian origin of this repetition (14, 42, 43). Colombia is a triethnic country, made up of a population of Native American, African, and European origin. Bogotá, the capital of Colombia, has a typical multiple ancestry population, showing a high proportion of people of European ancestry, followed by Native American and African (42). The higher frequency of Amerindian genetic markers presents a coherent result with a low frequency of repetition. This study provides an initial report of the frequency of expansions of hexanucleotide repeats in C9ORF72 in patients with FTD in the Colombian population and paves the way for further study of the possible genetic causes of FTD in Colombia.

## Data Availability Statement

The original contributions presented in the study are included in the article/Supplementary Material, further inquiries can be directed to the corresponding author/s.

## Ethics Statement

The studies involving human participants were reviewed and approved by Pontificia Universidad Javeriana, Facultad de Medicina. The patients/participants provided their written informed consent to participate in this study.

## Author Contributions

AL-C and MV-R: study concept development and study design. AL-C, MV-R, and DM: testing and data collection. AL-C, MV-R, EG-C, and IZ: data analysis and interpretation. AL-C, MV-R, and IZ: manuscript drafting and provision of critical reviews. All authors have participated in the work and approve the final version of the manuscript for submission.

## Conflict of Interest

The authors declare that the research was conducted in the absence of any commercial or financial relationships that could be construed as a potential conflict of interest.

## Publisher's Note

All claims expressed in this article are solely those of the authors and do not necessarily represent those of their affiliated organizations, or those of the publisher, the editors and the reviewers. Any product that may be evaluated in this article, or claim that may be made by its manufacturer, is not guaranteed or endorsed by the publisher.
